# Bird diversity and abundance in organic and conventional apple orchards in northern Japan

**DOI:** 10.1038/srep34210

**Published:** 2016-09-28

**Authors:** Naoki Katayama

**Affiliations:** 1 Biodiversity Division, Institute for Agro-Environmental Sciences, NARO, 3-1-3 Kannondai, Tsukuba-shi, Ibaraki 305-8604, Japan

## Abstract

Many studies have investigated the benefits of agri-environmental schemes, such as organic farming, on biodiversity conservation in annual systems, but their effectiveness in perennial systems is less well understood, particularly in bird communities in temperate regions of Asia. This study examined the effects of organic farming practices on species richness and abundance of breeding birds in apple orchards in northern Japan. Bird counts were conducted in six pairs of organic and conventional orchards during the breeding season in April and May 2015. The total species richness of birds, estimated by sample- and coverage-based rarefaction and extrapolation curves, was greater in organic orchards than in conventional orchards. Among the three dietary guilds (insectivore, granivore, and omnivore), only insectivorous species were more abundant in organic orchards than in conventional ones. This study offers the first quantitative evidence that organic farming can be beneficial for enhancing the diversity of birds, particularly of insectivores, in fruit orchards in Japan.

With human populations and per capita consumption increasing globally, food production will need to increase by 70–100% in the next few decades^1^, which could lead to further agricultural expansion and intensification^2^. This has raised the question of whether food production and biodiversity conservation should be separated and intensive farming used (known as “land sparing”^3^) or whether it should be integrated on the same land and less intensive agriculture used (“land sharing” such as wildlife-friendly farming^3^ and high nature value farmland^4^). Recent empirical research has increasingly suggested that a land-sparing strategy achieves larger population sizes of many species than a land-sharing strategy and thus is a better way to balance food production and biodiversity, particularly in tropical regions (e.g., refs 5, 6, 7).

However, these studies often overlook the functional role of biodiversity on farms, such as pest control and pollinator services, and thus agricultural sustainability^8^. In addition, the land-sparing and land-sharing strategies are not mutually exclusive, as shown by the wide array of such strategies used in Europe, and many conservationists believe that a combination of these strategies is needed for effective conservation of biodiversity and ecosystem services^9^,^10^,^11^. Thus, land sharing is likely also useful for achieving a better balance between food production and biodiversity, particularly in regions where land sparing is unrealistic for social reasons^12^ or the benefits of wildlife-friendly farming or high nature value farmland on biodiversity are clear^4^,^13^.

In the temperate regions of Europe and North America, the benefits of agri-environmental schemes such as organic farming for biodiversity have long been investigated^14^. Earlier reviews or meta-analyses showed that there was a tendency for organic farming to increase biodiversity as compared to conventional or intensive farming^15^,^16^,^17^,^18^. However, most of these studies were conducted in fields with annual crops such as cereals and vegetables, and there has been less focus on perennial crops such as fruit orchards and vineyards^19^,^20^. Because farming practices and habitat structures differ between annual and perennial systems, the response of biodiversity to agri-environmental schemes may also differ between these systems^21^.

Japan has a wide topographic gradient, with the landscape ranging from high mountains to coastal plains^22^. The country is mainly covered by forests (68.5% of the land area) and agricultural areas (12.4%; FAO, http://www.fao.org/countryprofiles/index/en/?iso3=JPN). Most protected areas in Japan lie within national parks, which are characterized by a high proportion of forest areas and low landscape heterogeneity, and thus can effectively conserve forest birds^23^. However, agricultural and grassland birds prefer agricultural areas with high landscape heterogeneity consisting of farmlands, secondary forests, grasslands, and creeks^23^, as represented by the traditional Japanese rural landscapes (known as Satoyama^24^,^25^). Because it is unrealistic to purchase such agricultural landscapes for conversion to protected areas in Japan, facilitating their sustainable use for farming and wildlife is a more realistic option^23^. In fact, the national and local governments in Japan have introduced agri-environmental schemes in which farmers are paid to modify their farming practices to provide environmental benefits (e.g., organic farming and reduced application of chemical pesticides)^26^. To date, although the effects of wildlife-friendly farming on biodiversity have been investigated in annual systems (i.e., rice fields^26^,^27^), few empirical studies have been conducted in fruit orchards. Filling this knowledge gap will advance our understanding of the development of robust conservation planning adapted for future changes in climate and land use.

This study focused on bird communities inhabiting apple orchards in Aomori prefecture, northern Japan. Birds are considered to be appropriate biodiversity indicators of farming practices^28^ and may act as biological pest control agents in orchards and vineyards^29^,^30^. This study provides the first quantitative report of how organic farming affects the species richness or abundance of birds compared with conventional agriculture in apple orchards in Japan.

## Results

**Figure 1:**
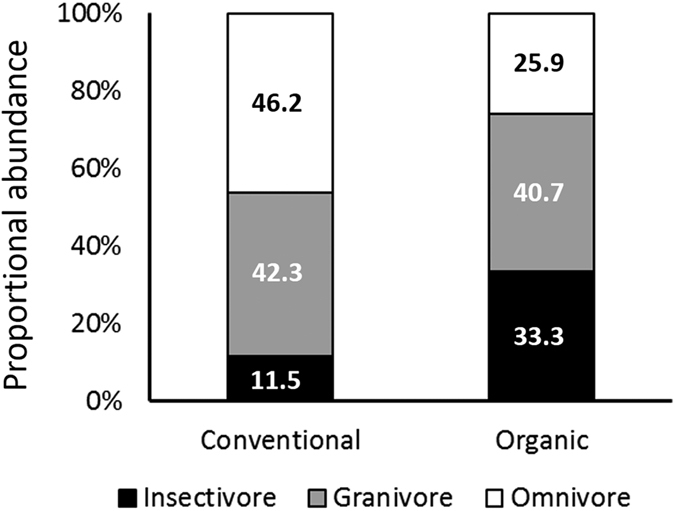
Proportional abundance of the three dietary guilds observed in conventional and organic apple orchards (calculated from data in Table 1).

In total, 13 bird species and 53 individuals were recorded in all the orchards (Table 1). Although the total abundance was nearly identical between the conventional and organic orchards, the proportional abundance of the three dietary guilds differed between the two systems: insectivores and omnivores were more and less abundant in organic orchards than in conventional orchards, respectively (Table 2; Fig. 1).

**Figure 2:**
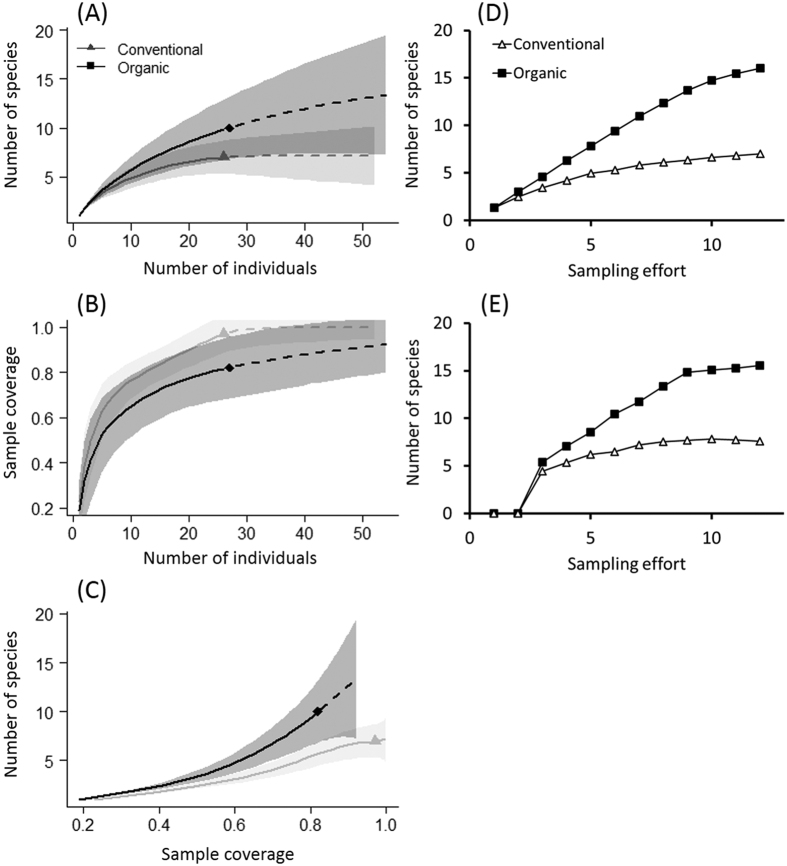
(**A**) Sample-based rarefaction and extrapolation for bird species richness in conventional and organic apple orchards. (**B**) Plot of sample coverage for rarefied samples as a function of sample size for individual birds. (**C**) Coverage-based rarefaction and extrapolation for bird species richness. (**A**–**C**) Solid and dashed lines are rarefaction and extrapolation (up to double the reference sample size), respectively, and the 95% confidence intervals were obtained by a bootstrap method based on 500 replications. (**D**,**E**) Estimated bird richness based on (**D**) Chao1 and (**E**) ACE. Sampling effort indicates the cumulative number of plots and surveys.

Sample-based rarefaction and extrapolation curves showed higher species richness of birds in organic orchards than in conventional orchards across a range of sample sizes, although their 95% confidence intervals overlapped (Fig. 2A). At a sample size of 26 individuals, species richness was estimated to be 7.0 and 9.8 in conventional and organic orchards, respectively. The sample completeness curve indicated that the sample completeness for the conventional orchards was higher than that for the organic orchards for any sample size, although the confidence intervals overlapped (Fig. 2B). When sample size in the conventional orchards was doubled from 26 to 52 individuals, the sample coverage increased from 97.1% to 100%. In the organic orchards, when sample size was doubled from 27 to 54 individuals, the coverage increased from 82.0% to 92.1%. Coverage-based rarefaction and extrapolation curves showed higher species richness of birds in organic orchards than in conventional orchards across a range of sample coverage, and their 95% confidence intervals overlapped slightly only when sample coverage was >85.0% (Fig. 2C).

The two non-parametric estimators, Chao1 and ACE, also showed higher species richness of birds in organic orchards than in conventional orchards across a range of sampling effort (Fig. 2D,E).

## Discussion

All of the rarefaction methods showed higher species richness in organic orchards than in conventional ones, but their 95% confidence intervals overlapped (Fig. 2). This is not surprising given the small sample size in this study. The higher sampling coverage and narrower confidence intervals of species richness in conventional orchards than in organic ones suggest that more of the species present were observed in conventional orchards due to lower species richness. If the sites had been surveyed more often, it is likely that the sample completeness curves would level off and the intervals become narrower, particularly for organic orchards, and the difference in species richness between orchard types would be more pronounced. However, the present results indicate that organic orchards supported more species of birds than conventional orchards.

I found a positive impact of organic farming on the diversity of bird communities, particularly of insectivorous species. This finding was consistent with studies conducted in fruit orchards in other areas (Europe^28^,^31^ North America^32^ and New Zealand^33^). In conventional apple orchards in Japan, synthetic chemical pesticides (e.g., broad-spectrum neurotoxic insecticides) are commonly used against several pest species such as peach fruit moth (*Carposina niponensis*) and aphids (http://www.jacom.or.jp/nouyaku/). These pesticides can affect birds directly by altering the central nervous system or indirectly by reducing available food resources^28^,^31^,^32^. In Japan, the intensity of pest control management is negatively correlated with the occurrence of macroinvertebrates such as carabid beetles, spiders^34^ and earthworms^35^, which are food sources for insectivorous birds. In addition to pesticide use, grass management might have affected the diversity and abundance of insectivorous birds. Although not significantly different, the grass was 5 cm taller in organic orchards than in conventional ones (Table 3). Caprio *et al*.^36^ reported that grass height was positively correlated with species richness and abundance of macropterous carabid beetles and ground hunting spiders in vineyards in Italy.

In contrast to the impact of conventional farming on insectivorous species, I did not find any evidence of a negative impact of conventional farming on granivorous or omnivorous species. Oriental greenfinch (*Chloris sinica*), the only granivore found in this study, mainly eats seeds on the ground during the breeding season^37^. In the study area, there was no clear difference in grass cover between conventional and organic orchards (Table 3), which may explain the lack of effect of organic farming on this species. For the omnivorous species, Eurasian tree sparrow (*Passer montanus*), brown-eared bulbul (*Hypsipetes amaurotis*), and white-cheeked starling (*Spodiopsar cineraceus*) are adapted to moderately to highly disturbed environments such as green space in urban areas^38^. Thus, it is possible that factors other than farming practices (e.g., distance to residential areas) affected their richness and abundance. Although not significantly different, conventional orchards were a little smaller than organic orchards (Table 3) and thus may be closer to urban green space such as hedgerows and gardens.

In contrast to species richness and composition, total abundance was similar between organic and conventional orchards. This finding suggests that these habitats were preferred by different species and thus habitat heterogeneity at the local and landscape scales, as represented by Satoyama landscapes^24^,^25^, is key to supporting high biodiversity at the regional level. More intensive surveys are needed to rigorously test this hypothesis.

In conclusion, this study showed the usefulness of organic farming, one of the agri-environmental schemes in Japan, for enhancing bird diversity. This finding supports the proposal by Naoe *et al*.^23^ that a combination of land-sparing and land-sharing strategies is useful for effective conservation of biodiversity and ecosystem services in Japan. In future studies, the effects of other local factors (e.g., orchard size, herbaceous ground cover, and tree density and diversity) and landscape structure (e.g., habitat diversity) on biodiversity should also be examined in both breeding and non-breeding seasons to understand what types of management should be included in agri-environmental schemes to reduce negative impacts on biodiversity^39^,^40^. In addition, understanding the role of birds as pest-control agents and enhancing their ability to hunt in orchards by using nest-boxes will be very important for sustainable food production in orchard systems^30^. Meeting these challenges will contribute to maintaining the balance between food production and biodiversity conservation.

## Methods

This study was carried out in accordance with the Act on Welfare and Management of Animals (Law No. 105, Japan) and the guidelines published by the National Institute for Agro-Environmental Science (http://www.niaes.affrc.go.jp/sinfo/animal_ex.html?160301). All experimental protocols were approved by the Institute for Agro-Environmental Sciences, NARO, and by farmers.

### Study area

**Figure 3:**
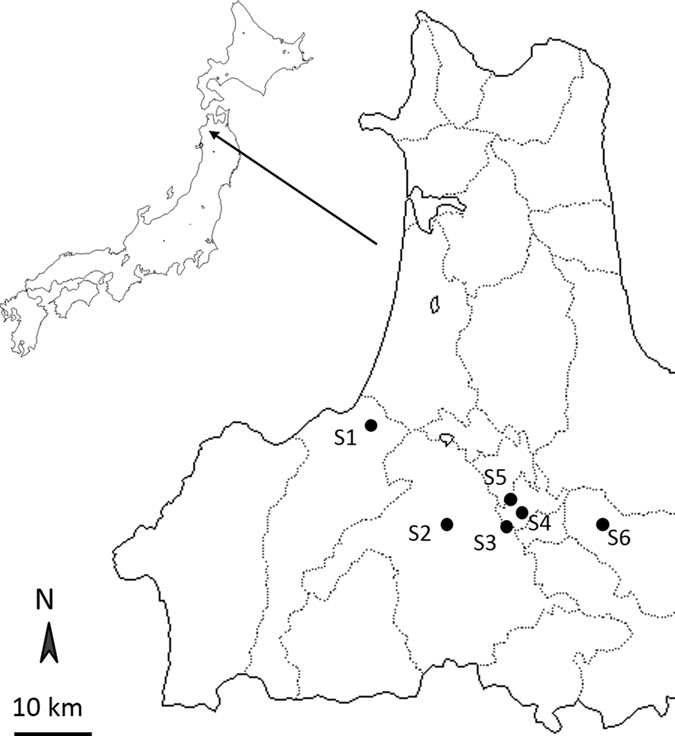
Locations of the six study sites (S1–S6) in Aomori prefecture, Tohoku region, northern Japan. At each site, one conventional and one organic apple orchard were chosen for bird surveys (see main text and Table 3 for more details on the sites). Maps of Japan and Aomori prefecture were derived from the Craft MAP website (http://www.craftmap.box-i.net/, accessed on April 10, 2016).

Field surveys were conducted in commercial apple orchards in Aomori prefecture, Tohoku region, northern Japan (Fig. 3). Aomori prefecture is the largest producer of apples in Japan; 468,000 tonnes of apples were harvested from 20,000 ha in 2014 (Statistics Bureau, http://www.e-stat.go.jp/SG1/estat/List.do?lid=000001139363). Commercial apple production started in the 1870s using cultivars introduced mainly from the United States^35^,^41^. Mean annual temperature and annual precipitation in the area from 1980 to 2010 were 10.2 °C and 1183 mm, respectively. Major land uses in Aomori prefecture are forest (65.7%), urban areas (17.4%), and farmland (16.3%).

Six sites were chosen within the study area, and one pair of conventional and organic orchards was chosen at each site. The sample size was limited because only 24.5 ha of organic orchards existed in Aomori prefecture in 2015 (Ministry of Agriculture, Forestry and Fisheries, http://www.maff.go.jp/j/jas/jas_kikaku/yuuki.html). The distance between the two orchards within a site was <1 km (mean = 0.63 km, SD = 0.41) to minimize the effects of surrounding landscape structure on birds in each orchard. The distance between each of the six sites was >10 km (mean = 13.5 km, SD = 8.7) to minimize the effects of spatial autocorrelation within each site. Although detailed information on pest control management in the conventional and organic orchards was not gathered in this study, conventional apple orchards are generally sprayed with fungicides, broad-spectrum insecticides (e.g., pyrethroid and neonicotinoid), and acaricides and chemical fertilizers are used^42^,^43^,^44^. Organic apples are grown with microbial inoculants and other non-pesticide measures and organic fertilizers, and are certified as organic products by the Ministry of Agriculture, Forestry and Fisheries (http://www.maff.go.jp/e/jas/). Other characteristics such as orchard size and grass cover and height, the latter two of which were recorded just after the end of bird counts (see below), did not significantly differ between the conventional and organic orchards sampled in this study (Table 3; note that latitude/longitude data are not shown to protect the personal information of the farmers).

### Bird counts

Bird counts were conducted during the breeding season in April and May 2015. Each orchard was visited two times by a single observer (NK) during favourable weather conditions (no rainfall and light wind) between 06:30 and 13:00. Dawn and dusk (before 05:00 and after 18:00) were avoided to minimize the effect of sampling time on bird detection in the study area with a large distance between sites. This seemed to be reasonable because there was no tendency for more individuals to be recorded earlier in the day (results not shown). The two fields at each pair of locations were sampled on the same day at each visit. One bird sampling point was placed at the centre of each orchard, and all birds heard or seen within a fixed radius of 50 m were recorded during 5 min. Care was taken to not count individual birds more than once. Birds that flew high above the sampling point were not included in the counts because it was uncertain whether these birds were inhabiting the study sites.

Each bird species was classified as insectivore, granivore, or omnivore following Takagawa *et al*.^45^, which is a compilation of life-history traits of birds in Japan. Three parameters were used to describe the bird community in each orchard: total species richness (number of species observed), total abundance (number of individuals), and abundance of the three dietary guilds.

### Statistical analyses

To estimate true species richness in the conventional and organic orchards and compare them, I employed a recent framework proposed by Chao *et al*.^46^, which unifies the sample-size-based approach of Colwell *et al*.^47^ and the coverage-based approach of Chao & Jost^48^. First, individual-based rarefaction and extrapolation curves of species richness were constructed for each orchard type (i.e., conventional or organic). Individual-based rarefaction curves were plotted against a given number of individuals chosen randomly from the observed samples until all individuals had been accumulated, and extrapolation curves were plotted to double the reference sample size^47^. Second, a sample completeness curve was constructed for each orchard type to link individual- and coverage-based sampling curves, up to double the reference sample size. Finally, coverage-based rarefaction and extrapolation curves of species richness were constructed for each assemblage. Coverage-based rarefaction curves were plotted against rarefied sample completeness, which is the estimated proportion of the total number of individuals in each orchard type represented by the species sampled^46^. Extrapolation curves were plotted to double the reference sample size. For all the types of rarefaction curves, I used 500 bootstrap replicates to estimate 95% confidence intervals. All estimates were obtained using the iNEXT package^49^ in R 3.2.3 software (R Project for Statistical Computing, http://www.r-project.org).

In addition to the above approach, I also employed two non-parametric, asymptotic species richness estimators for abundance-based data, Chao1 and ACE, by using EstimateS 9.1.0^50^. These estimators, which are widely used in biodiversity studies, are reported to not be greatly affected by the spatial scale of the sampling and thus perform well^51^.

## Additional Information

**How to cite this article**: Katayama, N. Bird diversity and abundance in organic and conventional apple orchards in northern Japan. *Sci. Rep.* **6**, 34210; doi: 10.1038/srep34210 (2016).

## Figures and Tables

**Table 1:** Identity and abundance of bird species observed in this study.

Dietary guild	Common name	Scientific name	Conventional orchards	Organic orchards	Total
Insectivore	Common reed bunting	*Emberiza schoeniclus*	0	3	3
	Barn swallow	*Hirundo rustica*	1	2	3
	Japanese tit	*Parus minor*	0	2	2
	Meadow bunting	*Emberiza cioides*	2	0	2
	White wagtail	*Motacilla alba*	0	1	1
	Bull-headed shrike	*Lanius bucephalus*	0	1	1
Granivore	Oriental greenfinch	*Chloris sinica*	11	11	22
Omnivore	Eurasian tree sparrow	*Passer montanus*	6	1	7
	Chestnut-cheeked starling	*Agropsar philippensis*	0	4	4
	Carrion crow	*Corvus corone*	2	1	3
	Brown-eared bulbul	*Hypsipetes amaurotis*	2	0	2
	White-cheeked starling	*Spodiopsar cineraceus*	2	0	2
	Naumann’s thrush	*Turdus naumanni*	0	1	1
Total abundance			26	27	53
Total species richness			7	10	13

**Table 2:** The five parameters (mean ± standard deviation) describing bird communities observed in conventional and organic apple orchards (note: statistical tests were not performed for these observed values).

	Conventional (*N *= 6)	Organic (*N *= 6)
Total species richness	2.17 ± 1.17	2.33 ± 1.03
Total abundance	4.33 ± 2.73	4.50 ± 2.59
Abundance of insectivores	0.50 ± 0.84	1.50 ± 1.39
Abundance of granivores	2.00 ± 1.67	1.17 ± 0.98
Abundance of omnivores	1.83 ± 1.17	1.83 ± 1.83

**Table 3:** Orchard characteristics (mean ± standard deviation) in conventional and organic apple orchards.

	Conventional (*N *= 6)	Organic (*N *= 6)	*P* value^a^
Orchard size (ha)	0.34 ± 0.12	0.50 ± 0.21	0.08
Grass cover (%)	65.0 ± 8.4	58.3 ± 27.9	0.48
Grass height (cm)	10.8 ± 2.0	15.8 ± 7.4	0.17

^a^*P* value was calculated from paired *t*-tests.

## References

[b1] Godfray H. C. J. . Food security: the challenge of feeding 9 billion people. Science 327, 812–818 (2010). 20110467 10.1126/science.1185383

[b2] Tilman D., Balzer C., Hill J. & Befort B. L. Global food demand and the sustainable intensification of agriculture. Proc Natl Acad Sci USA 108, 20260–20264 (2011). 22106295 10.1073/pnas.1116437108 PMC3250154

[b3] Green R. E., Cornell S. J., Scharlemann J. P. & Balmford A. Farming and the fate of wild nature. Science 307, 550–555 (2005). 15618485 10.1126/science.1106049

[b4] Morelli F., Jerzak L. & Tryjanowski P. Birds as useful indicators of high nature value (HNV) farmland in Central Italy. Ecol Indic 38, 236–242 (2014).

[b5] Edwards D. P. . Wildlife-friendly oil palm plantations fail to protect biodiversity effectively. Cons Lett 3, 236–242 (2010).

[b6] Phalan B., Onial M., Balmford A. & Green R. E. Reconciling food production and biodiversity conservation: land sharing and land sparing compared. Science 333, 1289–1291 (2011). 21885781 10.1126/science.1208742

[b7] Chandler R. B. . A small-scale land-sparing approach to conserving biological diversity in tropical agricultural landscapes. Cons Biol 27, 785–795 (2013). 10.1111/cobi.12046 23551570

[b8] Tscharntke T. . Global food security, biodiversity conservation and the future of agricultural intensification. Biol Cons 151, 53–59 (2012).

[b9] Tryjanowski P. . Conservation of farmland birds faces different challenges in Western and Central-Eastern Europe. Acta Ornithol 46, 1–12 (2011).

[b10] Scariot A. Land sparing or land sharing: the missing link. Science 334, 593–594 (2013).

[b11] Fischer J. . Land sparing versus land sharing: moving forward. Cons Lett 7, 149–157 (2014).

[b12] Miyashita T., Yamanaka M. & Tsutsui M. H. Distribution and abundance of organisms in paddy-dominated landscapes with implications for wildlife-friendly farming In Social and ecological restoration in paddy dominated landscapes (eds. Usio N. & Miyashita T.) 45–65 (Springer, 2014).

[b13] Hodgson J. A., Kunin W. E., Thomas C. D., Benton T. G. & Gabriel D. Comparing organic farming and land sparing: optimizing yield and butterfly populations at a landscape scale. Ecol Lett 13, 1358–1367 (2010). 20825453 10.1111/j.1461-0248.2010.01528.x

[b14] Tscharntke T., Klein A. M., Kruess A., Steffan-Dewenter I. & Thies C. Landscape perspectives on agricultural intensification and biodiversity - ecosystem service management. Ecol Lett 8, 857–874 (2005).

[b15] Bengtsson J., Ahnstrom J. & Weibull A. C. The effects of organic agriculture on biodiversity and abundance: a meta-analysis. J Appl Ecol 42, 261–269 (2005).

[b16] Hole D. G. . Does organic farming benefit biodiversity? Biol Cons 122, 113–130 (2005).

[b17] Batary P., Baldi A., Kleijn D. & Tscharntke T. Landscape-moderated biodiversity effects of agri-environmental management: a meta-analysis. Proc Biol Sci 278, 1894–1902 (2011). 21106585 10.1098/rspb.2010.1923 PMC3097824

[b18] Tuck S. L. . Land-use intensity and the effects of organic farming on biodiversity: a hierarchical meta-analysis. J Appl Ecol 51, 746–755 (2014). 25653457 10.1111/1365-2664.12219 PMC4299503

[b19] Simon S., Bouvier J. C., Debras J. F. & Sauphanor B. Biodiversity and pest management in orchard systems. A review. Agron Sustain Dev 30, 139–152 (2010).

[b20] Rey P. J. Preserving frugivorous birds in agro-ecosystems: lessons from Spanish olive orchards. J Appl Ecol 48, 228–237 (2011).

[b21] Bruggisser O. T., Schmidt-Entling M. H. & Bacher S. Effects of vineyard management on biodiversity at three trophic levels. Biol Cons 143, 1521–1528 (2010).

[b22] Yamaura Y., Amano T., Kusumoto Y., Nagata H. & Okabe K. Climate and topography drives macroscale biodiversity through land-use change in a human-dominated world. Oikos 120, 427–451 (2011).

[b23] Naoe S. . Identifying priority areas for national-level conservation to achieve Aichi Target 11: a case study of using terrestrial birds breeding in Japan. J Nat Cons 24, 101–108 (2015).

[b24] Katoh K., Sakai S. & Takahashi T. Factors maintaining species diversity in satoyama, a traditional agricultural landscape of Japan. Biol Cons 142, 1930–1936 (2009).

[b25] Katayama N. . Landscape heterogeneity-biodiversity relationship: effect of range size. Plos One 9, e93359 (2014). 24675969 10.1371/journal.pone.0093359 PMC3968173

[b26] Katayama N., Baba Y. G., Kusumoto Y. & Tanaka K. A review of post-war changes in rice farming and biodiversity in Japan. Agric Syst 132, 73–84 (2015).

[b27] Katayama N., Murayama H. & Mashiko M. The effect of organic farming on food intake and abundance of egrets and herons in rice fields. Jpn J Ornithol 64, 1–11 (in Japanese with English summary) (2015).

[b28] Genghini M., Gellini S. & Gustin M. Organic and integrated agriculture: the effects on bird communities in orchard farms in northern Italy. Biodivers Cons 15, 3077–3094 (2006).

[b29] Mols C. M. M. & Visser M. E. Great tits can reduce caterpillar damage in apple orchards. J Appl Ecol 39, 888–899 (2002). 10.1371/journal.pone.0000202 PMC1784073 17285148

[b30] Jedlicka J. A., Greenberg R. & Letourneau D. K. Avian conservation practices strengthen ecosystem services in California vineyards. Plos One 6, e27347 (2011). 22096555 10.1371/journal.pone.0027347 PMC3212556

[b31] Bouvier J. C., Ricci B., Agerberg J. & Lavigne C. Apple orchard pest control strategies affect bird communities in southeastern France. Environ Toxicol Chem 30, 212–219 (2011). 20928901 10.1002/etc.377

[b32] Fluetsch K. M. & Sparling D. W. Avian nesting success and diversity in conventionally and organically managed apple orchards. Environ Toxicol Chem 13, 1651–1659 (1994).

[b33] MacLeod C. J., Blackwell G. & Benge J. Reduced pesticide toxicity and increased woody vegetation cover account for enhanced native bird densities in organic orchards. J Appl Ecol 49, 652–660 (2012).

[b34] Funayama K. Influence of pest control pressure on occurrence of ground beetles (Coleoptera: Carabidae) in apple orchards. Appl Entomol Zool 46, 103–110 (2011).

[b35] Mochizuki T., Hanada S., Saitoh H. & Chiba S. Ecological study on the apple orchard soils contaminated by inorganic agricultural chemicals, 1: Effects of the contents of residual copper, lead and arsenate on the soil macrofauna of the apple orchards in Tsugaru district of Aomori Prefecture, Japan. J Sci Soil Manure 46, 45–50 (in Japanese) (1975).

[b36] Caprio E., Nervo B., Isaia M., Allegro G. & Rolando A. Organic versus conventional systems in viticulture: comparative effects on spiders and carabids in vineyards and adjacent forests. Agric Syst 136, 61–69 (2015).

[b37] Yoshikawa T. & Osada Y. Dietary compositions and their seasonal shifts in Japanese resident birds, estimated from the analysis of volunteer monitoring data. Plos One 10, e0119324 (2015). 25723544 10.1371/journal.pone.0119324 PMC4344244

[b38] Kobori H. . Citizen science: a new approach to advance ecology, education, and conservation. Ecol Res 31, 1–19 (2016).

[b39] Duarte J., Farfan M. A., Fa J. E. & Mario Vargas J. Soil conservation techniques in vineyards increase passerine diversity and crop use by insectivorous birds. Bird Study 61, 193–203 (2014).

[b40] Myczko Ł. . Effects of management intensity and orchard features on bird communities in winter. Ecol Res 28, 503–512 (2013).

[b41] Igarashi M., Hatsuyama Y., Harada T. & Fukasawa-Akada T. Biotechnology and apple breeding in Japan. Breeding Science 66, 18–33 (2016). 27069388 10.1270/jsbbs.66.18 PMC4780799

[b42] Funayama K. Unmown groundcover conserves adult populations of the predatory ground beetle Chlaenius micans (Coleoptera: Carabidae) in commercial apple orchards. Appl Entomol Zool 49, 183–187 (2014).

[b43] He Y.-H. . Oligo-DNA custom macroarray for monitoring major pathogenic and non-pathogenic fungi and bacteria in the phyllosphere of apple trees. Plos One 7, e34249 (2012). 22479577 10.1371/journal.pone.0034249 PMC3316626

[b44] Matsushita Y. . Community structure, diversity, and species dominance of bacteria, fungi, and nematodes from naturally and conventionally farmed soil: a case study on Japanese apple orchards. Org Agric 5, 11–28 (2015).

[b45] Takagawa S. I. . JAVIAN Database: a species-level database of life history, ecology and morphology of bird species in Japan. Bird Res 7, R9‐R12 (2011).

[b46] Chao A. . Rarefaction and extrapolation with Hill numbers: a framework for sampling and estimation in species diversity studies. Ecol Monog 84, 45–67 (2014).

[b47] Colwell R. K. . Models and estimators linking individual-based and sample-based rarefaction, extrapolation and comparison of assemblages. J Plant Ecol 5, 3–21 (2012).

[b48] Chao A. & Jost L. Coverage-based rarefaction and extrapolation: standardizing samples by completeness rather than size. Ecology 93, 2533–2547 (2012). 23431585 10.1890/11-1952.1

[b49] Hsieh T., Ma K. & Chao A. iNEXT: iNterpolation and EXTrapolation for species diversity. R package version 2.0.9 (2013).

[b50] Colwell R. K. EstimateS: Statistical estimation of species richness and shared species from samples. Version 9. (2013) Available at http://purl.oclc.org/estimates (Accessed: 2nd December 2015).

[b51] Hortal J., Borges P. A. & Gaspar C. Evaluating the performance of species richness estimators: sensitivity to sample grain size. J Anim Ecol 75, 274–287 (2006). 16903065 10.1111/j.1365-2656.2006.01048.x

